# Unravelling the Cerebellar Involvement in Autism Spectrum Disorders: Insights into Genetic Mechanisms and Developmental Pathways

**DOI:** 10.3390/cells13141176

**Published:** 2024-07-10

**Authors:** Marika Guerra, Vanessa Medici, Gina La Sala, Donatella Farini

**Affiliations:** 1 Department of Neuroscience, Section of Human Anatomy, Catholic University of the Sacred Hearth, 00168 Rome, Italy; marika.guerra@unicatt.it (M.G.); vanessa.medici@guest.policlinicogemelli.it (V.M.); 2 Institute of Biochemistry and Cell Biology, Italian National Research Council (CNR), 00015 Monterotondo Scalo, Italy; 3 Department of Biomedicine and Prevention, University of Rome Tor Vergata, 00133 Rome, Italy

**Keywords:** cerebellum, autism spectrum diseases, organoids, ASD-associated genes

## Abstract

Autism spectrum disorders (ASDs) are complex neurodevelopmental conditions characterized by deficits in social interaction and communication, as well as repetitive behaviors. Although the etiology of ASD is multifactorial, with both genetic and environmental factors contributing to its development, a strong genetic basis is widely recognized. Recent research has identified numerous genetic mutations and genomic rearrangements associated with ASD-characterizing genes involved in brain development. Alterations in developmental programs are particularly harmful during critical periods of brain development. Notably, studies have indicated that genetic disruptions occurring during the second trimester of pregnancy affect cortical development, while disturbances in the perinatal and early postnatal period affect cerebellar development. The developmental defects must be viewed in the context of the role of the cerebellum in cognitive processes, which is now well established. The present review emphasizes the genetic complexity and neuropathological mechanisms underlying ASD and aims to provide insights into the cerebellar involvement in the disorder, focusing on recent advances in the molecular landscape governing its development in humans. Furthermore, we highlight when and in which cerebellar neurons the ASD-associated genes may play a role in the development of cortico–cerebellar circuits. Finally, we discuss improvements in protocols for generating cerebellar organoids to recapitulate the long period of development and maturation of this organ. These models, if generated from patient-induced pluripotent stem cells (iPSC), could provide a valuable approach to elucidate the contribution of defective genes to ASD pathology and inform diagnostic and therapeutic strategies.

## 1. Introduction

Autism spectrum disorders (ASDs) are clinically heterogeneous neurodevelopmental disorders that predominantly occur in infancy or early childhood. They comprise two symptom domains: the social domain, which includes difficulties with social interactions and communication, the non-social domain, which is characterized by repetitive behaviors, and sensory perception abnormalities (as indicated in the *Diagnostic and Statistical Manual of Mental Disorders 5th Edn* DSM-5 [1] and in the International Classification of Diseases, 11th Revision, of World Health Organization 2019, available online: https://icd.who.int/en code 6A02). The etiology of ASD is multifaceted, with a significant genetic component that is exacerbated by environmental factors, including prenatal exposures such as valproic acid, which are known to increase ASD risk [2,3]. 

In particular, the genetic contributions to ASD are substantial, with heritability estimates of around 80% from twin and family studies [2,4,5], involving numerous mutations and genomic rearrangements. These genetic perturbations significantly impact neurodevelopmental pathways, particularly in the cortex during the second trimester of pregnancy and the cerebellum during the perinatal and early/postnatal period [6,7]. During the second trimester, the cortex, a complex neural network critical for cognition and social interactions, undergoes significant developmental processes such as neuronal proliferation, migration, and cortical layer formation. Genetic abnormalities in these processes are strongly associated with ASD symptoms that affect social communication and cognitive functioning. Conversely, the cerebellum, beyond its traditional motor functions, has emerged as a critical player in the development of behavioral circuitry. 

During the perinatal and early/postnatal periods, the cerebellum undergoes significant growth and structural refinement, involving granule cell (GC) migration, Purkinje cell (PC) dendritic arborization, and synaptogenesis. 

Genetic perturbations during this critical period can profoundly affect cerebellar circuitry and function, contributing to the neurodevelopmental alterations observed in individuals with ASD [7,8].

This review aims to explore the recent advancements in understanding the role of the cerebellum in ASD, focusing on the impact of ASD-associated genetic alterations on cerebellar development and their contribution to ASD pathogenesis. By examining experimental models that have elucidated the effects of these genetic factors, this review will shed light on the potential for future therapeutic interventions that target the intricate relationship between genetic influences and cerebellar development in ASD.

## 2. Syndromic and Non-Syndromic ASD

ASDs are categorized into syndromic and non-syndromic (idiopathic) types based on clinical criteria. These terms have recently been extended over to annotate genes (as in “syndromic” and “non-syndromic” genes for ASD), but like all classifications, it is simplistic [9,10].

Syndromic ASD, which accounts for less than 10% of clinical cases of autism [10,11], includes disorders characterized by both genetic anomalies and physical manifestations, such as Fragile X (FXS), *MECP2* duplication, Phelan–McDermid, and tuberous sclerosis complex (TSC) syndromes. 

These disorders are often characterized by significant genetic etiologies including chromosomal abnormalities like the 15q11-q13 duplication, 6p11.2 microdeletion, and 22q11.2 deletion syndrome, copy number variations (CNVs), or mutations in a single gene resulting in complex clinical profiles. The genetic underpinnings of syndromic ASD involve disruptions in transcriptional regulation and synaptic homeostasis, affecting the development of cognitive and behavioral circuits [11]. Notable genes involved include the *SHANK3* gene, mapped to chromosome 22, which encodes a postsynaptic membrane scaffolding protein necessary for the development, maturation, and plasticity of excitatory glutamatergic synapses [12]. *SHANK3* is deleted in Phelan–McDermid syndrome, which is characterized by severe intellectual disability, language delay, and autism [13]. This gene, along with *SHANK1* and *SHANK2*, is considered a high confidence gene associated with ASD in SFARI, the database with the most up-to-date information on all known human genes associated with ASD (https://sfari.org/). 

Studies in *Shank3*-deficient mice, which express this gene predominantly in the granule cell layer (GCL) of the cerebellum, highlight its critical role in sensory learning and its association with ASD [14]. These mice have normal cerebellar anatomy but show a significant reduction in the number of dendritic spines of PCs, suggesting an involvement of *SHANK3* in the development of mature neuronal connections [14]. 

Another significant gene, the X-linked *MECP2* gene, associated with *MECP2* duplication syndrome in boys [15] and Rett syndrome in girls [16], regulates gene expression through interactions with methylated DNA and plays a critical role in proper brain development from infancy through childhood [17,18]. Studies in mouse models have demonstrated that mutations in the MECP2 gene result in reduced cerebellar volume and impaired motor and associative behaviors. These findings underscore the critical role of MECP2 in cerebellar function and of the alteration of this brain area associated with ASD pathophysiology [19]. 

Non-syndromic ASD, typically referred to as classic autism, first described by Kanner in 1943 [20], is characterized by cognitive and behavioral symptoms only. The etiology of these forms of autism is unknown, with both rare and common variants contributing to their heritability [21,22]. Although individually these variants have a small effect, cumulatively they influence the risk of autism [23]. 

## 3. Genomic Complexity in ASD 

The complexity of the gene network associated with ASD has become apparent with the advances in molecular genetics and technologies for analyzing large genomic datasets. This complexity is evident in the diversity of genetic alterations, including copy number variants (CNVs), the primary form of inherited gene risk mutations for ASD, and *de novo* mutations, which play a significant role in ASD pathology. 

CNVs, which involve duplications or deletions of a portion of a chromosomal region, are the primary form of inherited gene risk mutations for ASD, although the consequences of these cytogenetic abnormalities are variable. They characterize most syndromic ASD, such as the Angelman syndrome (AS) [24], which is caused by a 15q11.13 deletion and accompanied by a deficit in the maternally inherited *UBE3A* gene. *UBE3A* is critical for protein degradation by the proteasome, and its deficiency at the embryonic ages leads to an accumulation of *UBE3A* targets with the disruption of specific brain functions. The gene is expressed preferentially in distinct brain areas, including PCs in the cerebellum, and many dysregulated pathways have been described in the cerebellar mouse model of the syndrome [25]. Specifically, an imbalance of mTORC1/mTORC2 signaling and oxidative stress in PCs, in addition to an abnormal tonic inhibition in GCs linked to reduced degradation and activity of the GABA transporter 1 (GAT1), have been associated with gait dysfunction and ataxia in AS mice [26,27].

In addition to common and rare CNVs, ASD patients frequently exhibit *de novo* structural genomic mutations that differ significantly from those of neurotypical individuals. 

These mutations primarily affect chromosomal regions linked to ASD-related genes, as detailed in a review by Vicari et al. [28]. 

The largest genome-wide association study (GWAS), involving 18,000 ASD patients and 28,000 controls from the Danish population, demonstrated the importance of single nucleotide polymorphisms (SNPs) in five ASD-specific-loci, predominantly located in regulatory regions crucial for human cortical development [29]. To expand the sample sizes of GWAS, the SPARK dataset (https://sparkforautism.org/) was utilized, resulting in the identification of two loci with novel variants [30]. The majority of the identified variants were found to be enriched in enhancers [31], with a particular focus on cerebellar enhancers at different developmental stages [32].

Among the predicted target genes of these enhancers, *PAX6*, *TCF4* and *ZMIZ1* have been annotated in the SFARI database. *PAX6* encodes a transcription factor that is crucial for cerebellar development, and its deletion in mice leads to aberrant growth of the glutamatergic neurons as GCs [33]. 

Over the past decade, genetic laboratories and consortia, using whole exome sequencing (WES), have identified rare inherited and *de novo* variants within the coding regions of the genome that are associated with ASD risk. These variants, predominantly loss of function (LoF) or insertion/deletion mutations, have a significant impact on the encoded proteins compared to common variants [34,35].

Using validated statistical approaches, such as TADA [36], susceptibility genes associated with ASD have been identified with high confidence (false discovery rate (FDR) threshold of <10%) [21,23,37]. The number of such genes increases with the number of samples sequenced. 

Data from a WES analysis of approximately 12,000 ASD patients from the Autism Sequencing Consortium (ASC) [38] revealed coding mutations in 102 autosomal genes [39], including 30 novel genes [21,34,39]. By increasing the number of individuals studied from the SPARK Consortium and SFARI Simons Simplex Collection (SSC), additional new genes have been identified [40,41]. 

A study by Trost et al. [42], which involved more than 7000 individuals with autism and 13,000 family members, identified 134 genes through whole genome sequencing (WGS). Of these, 67 were novel genes beyond complex DNA rearrangements such as tandem repeat expansions. 

A functional networks analysis of ASD risk genes reveals their involvement in specific biological processes such as gene expression regulation, neuronal communication, migration, and proliferation, all of which are important for neuronal development [21,34,39,40,41,42].

The WGS approach has shifted attention to the regulatory regions of genes and to non-coding genes such as lncRNA or miRNA [43]. The importance of these genomic studies is illustrated by the hyperexpansion of a CGG repeat in the 5′ untranslated region (UTR) of the *FMR1* gene of the FXS [44]. The hypermethylation of the repeats in the promoter region results in the silencing of the gene and the absence or greatly reduced synthesis of FMRP. FMRP is critical for normal brain function, particularly during specific periods of embryonic and early postnatal development [45,46]. It is responsible for the localization, stabilization, and translation of many brain mRNAs, including those encoding synaptic and ion channel proteins. 

In FXS, the most apparent dysfunction relates to motor behaviors like tremor and ataxia [47]. Alterations in the structure and function of the cerebellum have been observed in FXS patients. Studies in a mouse model have demonstrated altered synaptic plasticity and reduced spontaneous activity and excitability of PCs [48]. Interestingly, global *Fmr1^KO^* mice showed that in restoring *Fmr1* expression solely in PCs, an improvement in behavioral deficits was obtained, underscoring the pivotal role of the cerebellum in ASD behaviors [49]. 

All genetic approaches relevant to the identification of candidate gene variants associated with ASD require well-defined statistical applications to generate a significant gene list that can be refined and extended by gene ontology (GO) enrichment analysis to categorize the genes and by the establishment of a protein–protein interaction (PPI) network. One challenge lies in the retrieval of gene lists from different studies and databases, which often exhibit discrepancies. This is partly due to the heterogeneity of the samples analyzed and the statistical approaches employed. To consolidate evidences from different sources, we combined the 232 genes classified as high-confidence ratings for ASD (score of 1) in the SFARI gene database (https://gene.sfari.org/database/human-gene/ accessed on 5 July 2024), the 162-gene list from the SPARK Committee (http://sparkforautism.org/portal/page/spark-gene-list/ accessed on 5 July 2024), the 102 genes identified by Satterstrom and colleagues [39] starting from ASC, the 185 genes identified from four independent cohorts [41], and the 135 genes identified by the Autism Speaks Genomics Program (MSSNG, https://research.mss.ng) [42]. 

**Figure 1:**
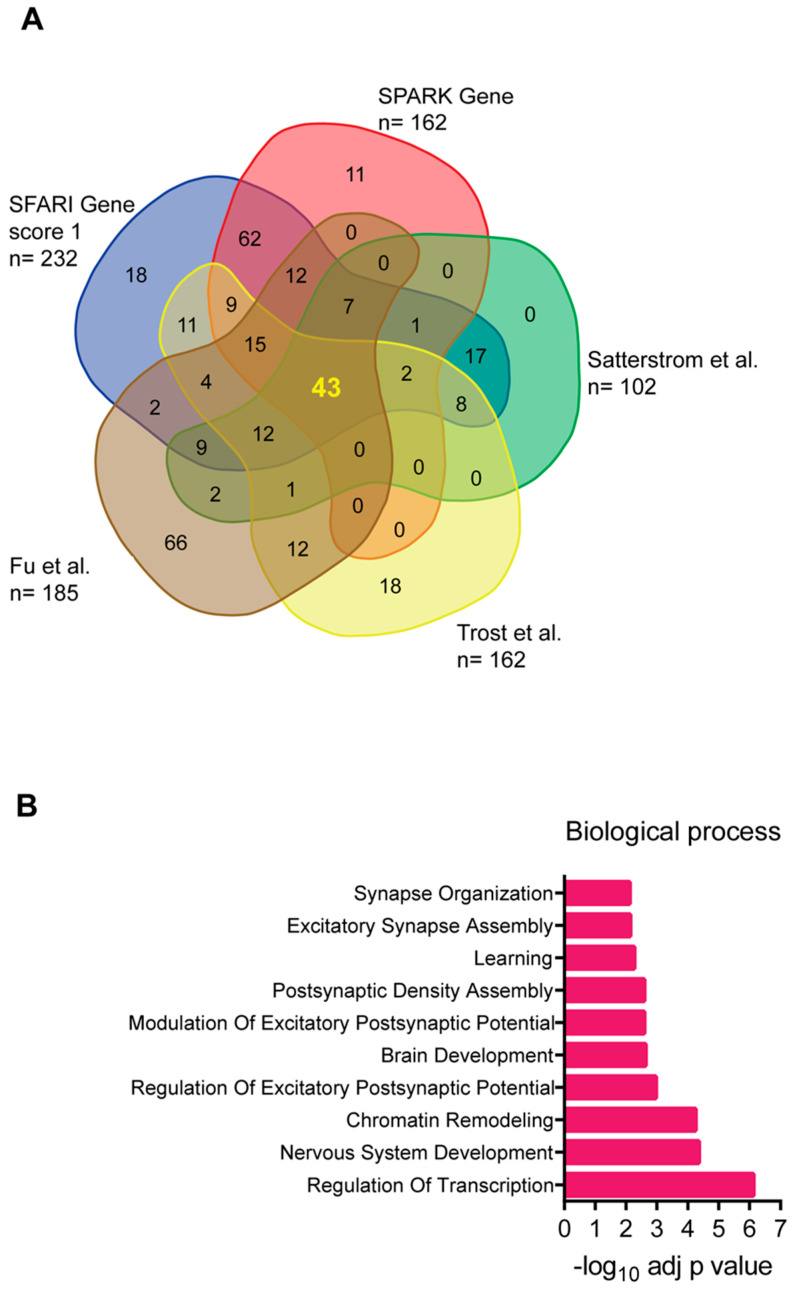
(**A**) Venn diagram showing the 43 ASD-associated genes obtained by overlapping the gene list from the different genomic databases and the author’s studies mentioned in the text [39,41,42]. (**B**) Gene ontology biological process of the 43 ASD-associated genes. Each term is expressed as −log_10_ of the adjusted *p*-value (Benjamini and Hochberg).

By overlaying the different sources, a core set of 43 genes was identified that consistently appeared across the datasets (Figure 1A). These genes are listed in Table 1. Many of the genes did not yield the highest SFARI gene score for ASD association. Further analysis through GO functional enrichment highlighted their involvement in crucial neural functions, including gene expression regulation and synaptic communication (Figure 1B). This suggests that they may have potential translational medical applications.

## 4. Neuropathological Mechanism of ASD

The identification of predisposing genes does not immediately translate into a mechanistic understanding of ASD. The critical questions of where, when, and in which cell types neurophysiology is altered for the development of this brain disorder remain unanswered. 

A comprehensive analysis of RNA-seq data from the Genotype-Tissue Expression (GTEx) resource (https://gtexportal.org) revealed that the strongest expression was observed in the developing cortex and cerebellar hemisphere [39,50]. 

Furthermore, the transcriptomic landscapes of ASD have been extensively studied through analyses of post-mortem brain tissue. The differential gene expression (DEG) between ASD samples and controls reveals a significant downregulation of genes related to synaptic signaling and plasticity. Additionally, there is a notable upregulation of microglial, astrocyte, and neural immune genes, which suggests a potential link between ASD and neuroimmune dysfunction [43,51,52]. Genetic studies have demonstrated that immunogenic factors involved in autoimmunity and allergic responses may have a regulatory function in both the mature and the developing brain and that these factors are correlated to autistic-like traits [53,54]. The most significant unanswered question arising from these studies is whether the immune dysfunction is a primary cause or a consequence of the underlying disorder.

Although advances in genomic and transcriptomic analysis have increased our knowledge of the biological basis of ASD risk, few studies have investigated the proteomic network in this pathology. Several biochemical approaches have been employed to elucidate the ASD proteome as reviewed by Murtaza et al. [55]. An early proteomics analysis of post-mortem ASD prefrontal cortex and cerebellum samples revealed differential protein expression. Specifically, those associated with myelination, synaptic function, and energy metabolism were decreased in the cortex, while their expression was increased in the cerebellum. This suggests a perturbation in functional connectivity in both regions [56]. Recent studies have demonstrated significant proteomic differences in the occipital cortex and cerebellum between individuals with autism ASD and those without the condition. These differences have been observed in various biological processes, including alterations in synaptic scaffolding, glutamatergic transmission, calcium signaling, and axonal cytoskeletal proteins [57]. These findings align with genetic and transcriptomic studies.

In addition to differential protein expression analysis, the identification of biologically relevant PPI networks and signaling pathways is crucial for elucidating pathogenic processes underlying ASD. However, the PPI networks built from ASD risk proteins through public databases may not accurately represent the active networks in the brain. Efforts to construct more specific neural “interactomes” using cultured neurons and hiPSC-derived neuronal models have begun to reveal the functional roles of ASD-related proteins in the frontal cortex and cerebellar hemispheres [55,58]. 

## 5. Cerebellar Involvement in ASD

Over the past decade, a few key characteristics of ASD have been identified. These include the involvement of specific brain regions, collectively referred to as the “social brain” and those closely associated with it. These include the prefrontal and occipital cortex, amygdala, hippocampus, limbic system, and dopaminergic areas. The cerebellum, which is traditionally associated with the modulation of motor functions, has been demonstrated to play a pivotal role in cognitive circuits and complex behaviors [59,60,61]. The “cognitive” functions of the cerebellum depend on its reciprocal connections with the cortex, which are established during development, and on the processing of the signals exchanged between different cortical regions. For a comprehensive review of the cerebellar connectivity within the “social brain” network, see Stoodley and Tsai (2021) [62] and Mapelli et al. (2022) [63]. 

New research indicates that insults to the cerebellum during a specific developmental window, particularly in the perinatal and early postnatal period, may lead to alterations in cognitive processes that manifest later in life and underlie the clinical outcomes of ASD [7,8]. 

The developmental alterations associated with ASD in the cerebellum, particularly in the cytoarchitecture of this brain area, have been studied mainly in mouse models that mimic both syndromic and idiopathic ASD pathology. For a detailed description, see Mapelli et al., 2022 [63].

### 5.1. Morphological and Functional Cerebellar Alterations in ASD

Neuroimaging studies, histopathological and molecular analysis of post-mortem brains (reviewed in Fetit 2021 [64]), and functional analysis of cerebellar connectivity have revealed abnormalities associated with neurodevelopmental disorders such as ASD.

#### 5.1.1. Cerebellar Anatomy and Neuronal Specification 

**Figure 2:**
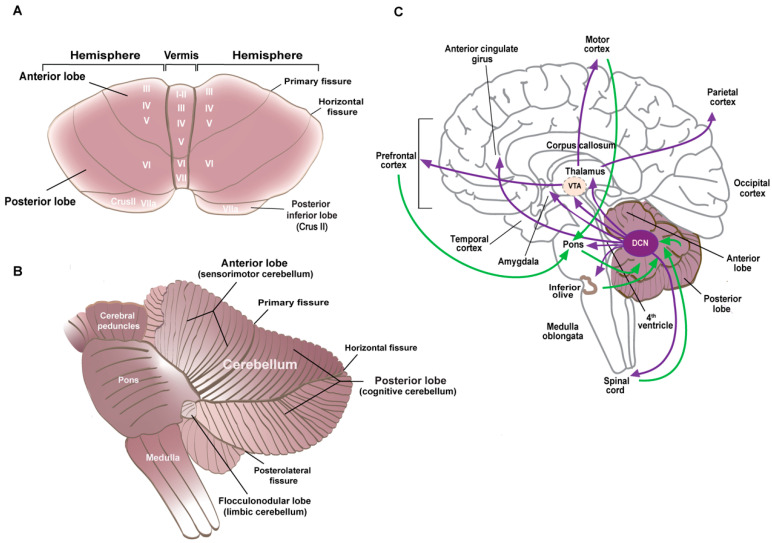
(**A**) Superior view of the isolated cerebellum. The lobes and the fissures that separate them are indicated. Roman numerals indicate the lobules. (**B**) Left lateral view of the cerebellum and brainstem. The functional divisions of the lobes are indicated with posterior lobules VI and VII in the vermis and the hemispheric extensions Crus I and Crus II forming the cognitive cerebellum. (**C**) Medial view of the brain with the efferent cerebellar connections indicated in purple and the afferents to the cerebellum in green. The deep cerebellar nuclei (DCN) project to the thalamus and midbrain, including the ventral tegmental area (VTA), amygdala, and anterior cingulate cortex. These regions then synapse with different cortical regions to form the cortico–cerebellar–cortical loops.

The cerebellum consists of a central part, the vermis, and two lateral extensions, the cerebellar hemispheres (Figure 2A). The folia divide the cerebellum anterior–posteriorly into three lobes, the anterior lobe (corresponding to the sensorimotor cerebellum), which is separated from the posterior lobe by the primary fissure, and the flocculonodular lobe below the posterolateral fissure (Figure 2B). The deep cerebellar nuclei (DCN: dentate, interpositus, and fastigial) consist of excitatory neurons that make polysynaptic cerebellar–cerebral projections through the thalamus and inhibitory neurons that project to the inferior olive (Figure 2C). 

**Figure 3:**
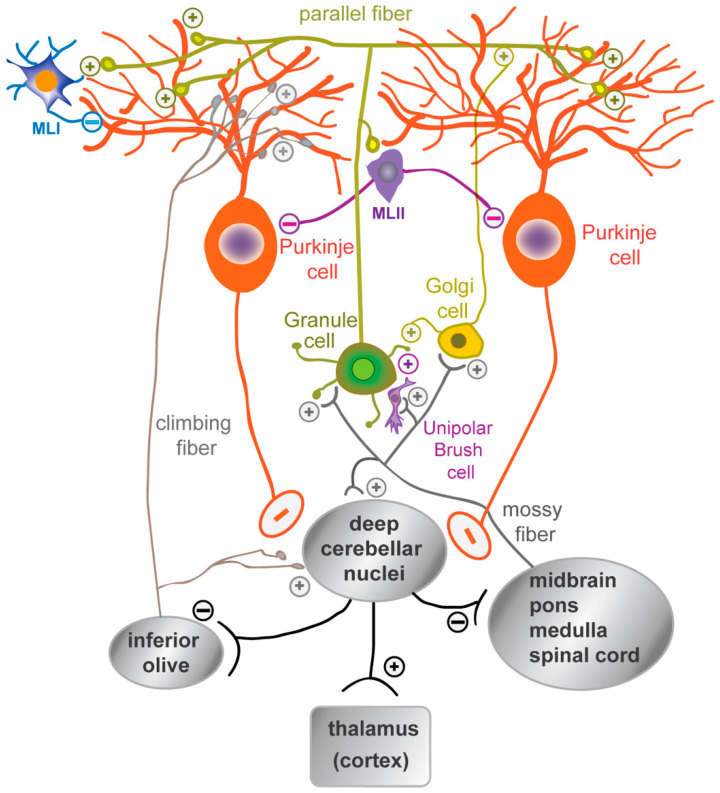
A schematic representation of the organization of mature cerebellar cells. The synaptic connections between the dendritic tree of the Purkinje cells and the parallel fibers of granule cells, as well as with the inhibitory molecular layer interneurons (MLI, MLII), are shown. Granule cell activity is modulated by Golgi cells and unipolar brush cells. The mossy fibers, originating from the brainstem and spinal cord nuclei, and the climbing fibers, originating from the inferior olive, are the excitatory afferent fibers that reach the cerebellar cortex. The mossy fibers regulate granule cell activity, while the climbing fibers synapse on the proximal dendrites of Purkinje cells.

The cerebellar neurons are organized into apparently simple circuits whose diversity and dynamics have recently been highlighted [65,66]. In the cerebellar cortex, a monolayer of inhibitory GABAergic PCs (Purkinje cell layer, PCL) represents the only output neurons projecting to the DCN, which in turn project to the brainstem, spinal cord, ventral tegmental area (VTA), and thalamus, and from there to the cortex. The DCN–thalamic projections cross the midline to the contralateral side in a manner analogous to the pontine–cerebellar afferents. PCs integrate direct excitatory afferent signals from the climbing fibers originating in the inferior olive and the mossy fibers from various nuclei (in the midbrain, pons, and spinal cord), which indirectly influence the PCs via the parallel fibers of the GCs. These axons establish excitatory synapses with the distal dendrites of PCs in the outer molecular layer (ML) of the cerebellar cortex (Figure 3). The refinement of the output signal is regulated by several populations of inhibitory interneurons, including Golgi cells in the GCL, molecular layer interneurons I and II (MLI, MLII), and Bergmann glial cells in the PCL, all of which play a crucial role in the proper development of the cerebellar cortex.

It is noteworthy that the development of the human cerebellum remains relatively understudied. This process extends from 30 days post conception (dpc) to the second postnatal year [67]. The prolonged period of its development renders it susceptible to insults, including genetic and environmental factors. 

Cerebellar neurons originate from radial glial progenitors in two primary zones of neurogenesis: the ventricular zone (VZ) and the rhombic lip (RL). Recently, the molecular characteristics and heterogeneity of progenitors in the two niches have been elucidated [68,69]. 

The VZ is responsible for the generation of all GABAergic populations during the embryonic period, including PCs. These neurons begin to differentiate at an early stage and can be identified as early as 47 dpc in a region that, similarly in the developing cortex, constitutes the sub-ventricular zone (SVZ) containing the mitotic progenitors [70,71]. 

**Figure 4:**
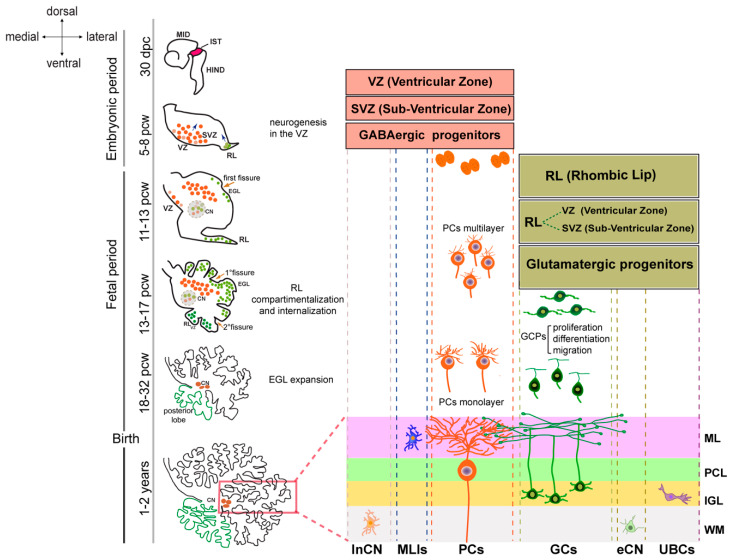
Human cerebellar development. The timeline of cerebellar development is shown on the left. The drawings show a 30 day post conception (dpc) embryo MID—midbrain, IST—isthmus, HIND—hindbrain, and cerebellar morphology at different stages of development. In the embryonic period, the cerebellar anlage is evident at 30 dcp. The progenitor zone of GABAergic neurons, including Purkinje cells (PCs), inhibitory neurons of the cerebellar nuclei (inCN), and molecular layer interneurons (MLIs), corresponds to a ventricular zone (VZ) and a sub-ventricular zone (SVZ). These regions expand until 8 postconceptional weeks (pcws) before regressing to a single cell layer. The glutamatergic progenitors of the excitatory neurons of the cerebellar nuclei (eCN), the granule cells (GCs), and the unipolar brush cells (UBCs) are born in the rhombic lip (RL), which begins its expansion in the fetal period. The external granular layer (EGL) appears at the end of the embryonic period and grows extensively throughout the fetal period due to the proliferation of granule cell precursors (GCPs). Between 13 and 14 pcw, the elongated RL divides into an RL-SVZ and an RL-VZ, which internalize and give rise to the neurons of the posterior lobe. The cortical layers in the mature cerebellum are marked (boxed area) and labeled on the right as follows: ML—molecular layer, PCL—Purkinje cell layer, IGL—internal granule layer, and WM—white matter.

During the fetal period, PCs migrate towards the outer surface of the cerebellar anlage, initially aggregating in multiple layers. The PCL, formed by a monolayer of PCs, is completed in the third trimester. After this, PCs undergo axon extension and dendritic tree expansion. In the meantime, the RL, which expands after VZ neurogenesis ceases, gives rise to all cerebellar glutamatergic neurons, including GCs. RL-derived progenitors migrate initially to give rise to neurons of the cerebellar nuclei and, subsequently, tangentially to the surface of the anlage, to form the external granule layer (EGL), which is populated by the immature GCs. In humans, the RL persists throughout gestation, undergoes internalization, and remains proliferative postnatally, contributing GC progenitors to the posterior vermis (Figure 4). 

During the third trimester, the cerebellum undergoes extensive growth due to the proliferation of the GCs, which migrate to become granule neurons of the internal granule layer (IGL). The migration of GCs is completed when the EGL disappears at the end of the first postnatal year.

During the postnatal period, cerebellar neurons undergo a maturation process that is characterized by the acquisition of intrinsic spontaneous activity, synaptic refinement, and pruning due to experience-induced plasticity and the activation of different signaling pathways. 

Advances in prenatal diagnosis have enabled the monitoring of cerebellar development during fetal life, thereby facilitating the early detection of malformations that were previously diagnosed in adolescents or adult patients.

#### 5.1.2. Cerebellar Morphology and Connectivity Abnormalities in ASD

Although there is conflicting evidence between different approaches, the cerebellum consistently emerges as one of the most affected brain areas in ASD, exhibiting abnormalities that also correlate with the severity of symptoms. 

The first description of hypoplasia of the posterior vermis was in a 21-year-old man diagnosed with autism [72]. This was subsequently confirmed by neuroimaging studies in children and adolescents [73,74]. A recent study employing magnetic resonance imaging (MRI) analysis revealed a statistically significant increase in lobular volume in several cerebellar regions, including the vermis, left and right lobule I–V, right Crus II, and right lobules VIIb and VIIIb, in children with ASD aged between 15 and 36 months when compared with both siblings and healthy controls [75]. 

Moreover, correlations were identified between morphological changes in the cerebellum and clinical scores based on cognitive, motor, and language functions. These studies confirm the previous observations [76] that altered cerebellar topography is associated with clinical deficits observed in children with ASD. Nevertheless, a more recent, larger study [77] did not yield compelling evidence for volumetric alterations in the cerebellum and its regions. This highlights the limitations of MRI-based methodologies, particularly when applied to small sample sizes, and the difficulties inherent in classifying lobules using disparate atlases as references. To accurately assess structural changes in the cerebellum in ASD, more sophisticated measures, such as local surface area and thickness of the cerebellar cortex, are required. 

The involvement of the cerebellum in ASD is also evidenced by the consequences of deficits that occur in this brain area, particularly at early stages of its development. This is exemplified by studies on preterm infants with isolated cerebellar injury. Limperopoulos et al. [78,79] have demonstrated that such injuries not only result in long-term motor deficits but also lead to the impairment of regional growth in the contralateral cerebral hemisphere. Additionally, they have shown that these injuries cause deficits in the development of cognition, communication, and social interaction. These deficits are indicative of contralateral cerebro-cerebellar projections, whereby the left cerebellar hemisphere forms circuits with right cerebral cortical regions that are involved in spatial processing, and the right cerebellar hemisphere interconnects with left cerebral language regions.

It has been demonstrated that perinatal damage to the cerebellum is associated with a high predicted risk of ASD and may result in the development of long-term ASD symptoms, including behavioral problems, deficits in social interactions, and in affect, attention, and other cognitive functions [8,78]. Wang et al. [7] proposed that the impact of early cerebellar damage on the neuropathology of ASD was due to the influence of this area on the development of cerebral cortical regions to which the cerebellum projects. 

The morphological alterations of the cerebellum are not exclusive to autism. This implies that the differences between neurodevelopmental disorders are likely to be found at the cellular and molecular levels. Post-mortem studies offer the opportunity to highlight the cellular changes in the autistic cerebellum. Among the most observed findings are a reduced number of PCs and alterations in their shape, particularly in the lateral hemispheres at Crus I and II [80,81]. This is accompanied by a reduction in GAD65 and GAD67, which catalyze the synthesis of GABA [80], and a reduction in the GABA receptor subunits, GABBR1 and 2 [82]. The reduction of GABAergic inputs from the cerebellum, in conjunction with alterations in GABA signaling, may contribute significantly to the hyperexcitability of cerebellar connections, particularly with the cortex. 

Cerebellar alterations have also been identified in animal models of idiopathic and monogenic ASD. For instance, rodents exposed to VPA during fetal development exhibit autistic features postnatally [83]. These mice exhibited morphological alterations in the posterior cerebellar lobules and a reduction in the number of PCs in these regions [84,85]. In addition, the BTBR inbred mouse line, which is known to display robust and consistent autism-relevant behaviors [86], exhibits delayed eyeblink conditioning accompanied by an expansion of the vermis and aberrant foliation in both the early postnatal and adult cerebellum [87,88]. BTBR mice exhibit a slight but significant reduction in PC density and a marked reduction in dendritic spines, the loss of which in ASD remains an open question. 

Cerebellar pathology has also been observed in individuals with FXS, characterized by atrophy of the vermis and reduced numbers of PCs [89]. A mouse model lacking *Fmr1* has been observed to exhibit alterations in the structure and plasticity of synapses on PCs [48,90]. 

Furthermore, atypical PC structure and function have been documented in the TSC disorder caused by mutations in TSC1 and TSC2 proteins, which negatively regulate the mTOR pathway [91]. A TSC1 mouse model of autism also exhibited similar cerebellar abnormalities [92]. 

In addition to the presence or absence of structural abnormalities, the cerebellum of individuals with ASD exhibit differences in functional connectivity. These differences frequently manifest as a reduction in the lateralization of the contralateral cerebellar-cerebral communications that are characteristic of motor, sensory, language, and cognitive functioning [93]. Deficits in sensorimotor behaviors, which emerge early in development, are a common core symptom in ASD patients. In a functional MRI (fMRI) study by Mostofsky and colleagues [94], a cohort of children were observed performing a finger motor task. It was observed that there was hyperactivation of premotor cortical regions, accompanied by decreased cerebellar activation in ASD patients. A reduction in the connectivity between the left anterior intraparietal lobule and the right Crus I has been observed in individuals with ASD during a visuomotor task. This alteration is more pronounced in children than in adolescents or adults, and it is associated with an increased severity of ASD symptoms [95]. Furthermore, atypical fMRI activation of cerebro-cerebellar circuitries has been observed in children with ASD during tasks involving motion perception or social interaction [96,97]. 

In contrast, Oldehinkel and colleagues [98] analyzed 20 well-characterized resting-state networks and demonstrated increased cerebellar connectivity between visual association, somatosensory, and motor networks in ASD subjects compared to typically developing subjects. This finding is consistent with those of other research groups [99,100]. It is of note that adolescents with ASD and infants at high familial risk for ASD exhibit hypoconnectivity between the right Crus I and language-related regions [101,102]. The findings on functional cerebellar connectivity alterations in ASD are dependent on the approach used (resting-state fMRI vs. task-based fMRI), the age of the subjects analyzed, and, most importantly, the inter-individual variance in cerebellar functional networks [103]. 

The findings collectively indicate a correlation between the morphological and functional cerebellar anomalies and the sensorimotor behaviors. Nevertheless, several questions remain unanswered. One such question is whether the sensorimotor deficit causes the reduced development of the cerebellum and thus a reduced number of functional cells, or whether the reverse is true. 

### 5.2. ASD Susceptibility Genes and Cerebellar Dysregulation

The complex interactions between the cerebellum and the brain areas involved in ASD, which undergo development and maturation during the first years of life, offers insights into why insults to the cerebellum during this developmental period can result in more severe behavioral and cognitive impairments than those observed in children and adults. 

Nevertheless, the molecular mechanisms underlying the link between the cerebellum and ASD remain unclear. To gain further insight, the use of mouse models has been invaluable. 

The transcriptional program governing mouse cerebellar development is highly dynamic and includes modulation of ASD-associated genes as evidenced by the significant overlap with genes dysregulated in human ASD brains and present in the SFARI and Autism Gene databases (AutDB, http://www.mindspec.org/autdb.html accessed on 5 July 2024) [84]. 

During the maturation of the mouse cerebellum, 162 ASD-associated genes are expressed, which significantly fall into functional processes that are frequently dysregulated in ASD. The most significant categories were related to neurogenesis, behavior, synapse maturation, and function, with a specific enrichment observed in genes related to the synapse involving the parallel fibers of GCs and PC dendrites [84]. Furthermore, an analysis of the transcriptome of mouse PCs, laser-captured at different developmental stages, confirmed the expression of ASD-associated genes in these neurons. This finding underscores the vulnerability of PCs during this early developmental stage [104]. It is of note that the gene expression profile of PCs differed from that observed in the cortex during the same developmental period, thus highlighting the unique molecular features of these cerebellar neurons [104].

In contrast to mice, the transcriptional landscape of the human cerebellum is underrepresented in transcriptomic studies of the developing brain. This limitation diminishes the statistical power of ASD gene enrichment analysis. Moreover, the gene expression patterns observed in the human cerebellum diverge from those observed in the mouse during development [69,105]. 

A molecular program that characterizes progenitors and differentiating cells in the human cerebellum, particularly in the fetal period, has been recently highlighted using single-cell and spatial transcriptomic approaches [68,69,105]. These approaches have revealed the existence of a complex molecular landscape underlying the differentiation of cerebellar cells. These studies demonstrated that cerebellar neurons generated during early development exhibit spatial and molecular heterogeneity, which will lead to the formation of distinct neural projections and connections in the adult organ. 

By employing single-cell chromatin accessibility methods, researchers have elucidated the cis-regulatory elements and transcription factors that are instrumental in determining the fate of distinct cerebellar cells [69,105]. This approach has revealed that PCs and GCs exhibit spatial heterogeneity and are determined in a hierarchical manner, each with a distinct molecular signature. For instance, at post-conception week (pcw) 10, all progenitor cells in the ventricular zone (VZ) express *RORA*, a type of retinoic acid-related orphan receptor. However, by postnatal day 14, only a subset of *RORA*-positive PCs that migrated to the outer edge of the cerebellar cortex expressed *RORB* [69]. 

Interestingly, *RORB*, which plays a role in regulating PC differentiation and maturation, is among the 43 ASD-associated genes (Figure 1, Table 1) and is characterized by rare single mutations [34,39]. A consensus sequence analysis of the 286 potential target genes expressed by PCs at the beginning of differentiation [69] identified 13 genes, including *RORB*, that were highly significant for ASD in the SFARI database (Table 2). These genes, including *SCN2A*, *SHANK2*, *GRIN1*, and *NRXN1*, and *3*, play a critical role in axonogenesis, synaptic assembly, and signaling (Table 2). Although this in silico analysis requires further experimental confirmation, it suggests a possible involvement of RORB in ASD neuropathology.

The importance of accurate molecular and regional PC differentiation in establishing the correct connections within the “cognitive brain” is underscored by the observation that ASD-associated genes are prominently expressed in prenatal PCs [68,69,105]. It is not surprising that these genes are classified into functional categories that mirror those observed in the cortex: chromatin organization and neuronal development [68,106]. Furthermore, it has been observed that PCs display an enrichment of SNPs in both coding and non-coding regions of genes associated with ASD, providing additional confirmation of their involvement in the pathogenesis of the disorders [69]. 

During the transcriptionally mapped fetal period, when ASD-associated genes are highly expressed, neurogenesis takes place in the cerebellar nuclei, and PCs emerge as the most predominant neuronal population. These cells migrate and initiate axonogenesis before organizing into a monolayer. Meanwhile, GCPs migrate to the cerebellar cortex to form the EGL before initiating their expansion phase, which characterizes the postnatal period. The lack of functional and molecular changes in highly proliferative and therefore vulnerable GCPs and GCs is intriguing. GCs play a crucial role in the proper maturation of PCs and receive mitogenic signals from them, such as SHH, and are driven by PCs in their migration from the EGL to the IGL. It can be hypothesized that ASD-mutated genes may indirectly influence the differentiation of GCs due to the close functional relationship between PCs and GCs. 

By examining the gene expression profiles of ASD-associated genes during cerebellar development, it became clear that they are expressed at higher levels during prenatal development compared to postnatal development [105]. However, the cerebellar maturation process during the neonatal and early postnatal periods is a complex one. The number of inputs undergoes a rapid change, and synaptogenesis occurs, particularly the formation of synapses between parallel fibers and PCs. Furthermore, the activity-dependent maturation of climbing fibers involves the pruning and relocation of these fibers from the soma to the dendrites of PCs. 

Brandenburg and colleagues have demonstrated transcriptomic differences in PCs that were laser-microdissected from post-mortem cerebella of young ASD patients (mean age 19 years) compared to normal tissue [107]. Of the 427 differentially expressed genes, the majority were downregulated compared to controls. These genes produced protein–protein interaction clusters related to neurogenesis, cell adhesion and migration, and cell–cell communication. This finding confirms an altered developmental trajectory of PCs in ASD pathology. 

Ament and colleagues recently proposed a single-cell transcriptomic atlas of the human cerebellum during childhood (one to five years), which highlights the heterogeneity of PCs [108].

These neurons are typically identified by the expression of *ALDOC/ZEBRIN2*, and each of the two differentiated populations expresses specific genes, thus indicating functional differences between stripes and representing specific developmental trajectories. While the distribution of spatial stripes and the function of the two main subtypes of PCs are known in rodents, the heterogeneity of PCs in humans is still being elucidated. Ament and colleagues confirmed that risk genes for ASD are enriched in prenatal PCs and decline during early childhood. The authors observed that PCs, other than Golgi interneurons, are particularly susceptible to inflammation during childhood, which is a well-known risk factor for ASD [109]. 

In post-mortem cerebella exhibiting inflammatory conditions, synaptic genes associated with ASD, such as *SHANK1*, are downregulated in PCs. This suggests a premature defective in their maturation, which could result in the altered connectivity typical of ASDs. The transcriptomic alterations associated with early-life inflammation appear to rescue the GCs, even though they are the most abundant and morphologically simple neuronal population in the cerebellum. These findings corroborate the hypothesis that genetic and environmental factors converge to influence the development of cerebellar neurons, potentially contributing to the etiology of ASD. However, it is not clear how the ASD risk genes affect the development and functionality of PCs. Mouse models have been used to induce PC-specific deletion of a number of these genes, which has revealed the effects on specific behaviors indicative of social impairment. A variety of behavioral tests are employed to assess social, cognitive, and repetitive processing in mice. Most of the tests can be conducted exclusively on adult animals, apart from ultrasonic vocalization, which can be evaluated in postnatal young animals. Most behavioral paradigms are affected in PC-conditional knockout (KO) animals when the deleted genes are involved in synaptic structure or plasticity. For example, *Shank2^KO^* mice do not exhibit motor impairment, but they display deficits in motor learning, as well as in social interaction in the three-chamber test, and in specific repetitive behaviors [110,111].

## 6. ASD Models to Highlight Pathology in the Cerebellum

The study of the cerebellar development is crucial for the understanding of its role in neurodevelopmental disorders, but access to human cerebellar tissue is challenging. In addition to animal models, *in vitro* models and protocols can be used to differentiate cerebellar neurons and generate a 3D self-organizing organoid from hiPSCs. 

The differentiation of hiPSCs into cerebellar neurons is intended to reproduce the neurogenesis of the cerebellum, which is a vulnerable period for the risk of ASD. Previous studies have primarily focused on the generation of immature PCs in hiPSC cultures, which can develop a low-branched dendritic tree after 90 days when co-cultured with mouse cerebellar cells [112]. 

Furthermore, excitatory neurons derived from RL have been differentiated using a chemically defined medium, resulting in the generation of GCs after 48 days *in vitro* [113]. A model of ASD using hiPSC-differentiated PCs was obtained from individuals diagnosed with TSC and ASD, carrying a mutation in the *TCS2* gene that causes hyperactivation of the mTORC1 signal [114]. *TSC2*-deficient hiPSCs exhibit differentiation deficits, resulting in PCs with increased soma size, synaptic alterations, and reduced excitability. Additionally, the neurons exhibited indications of autophagy activation and oxidative stress, which ultimately resulted in cell death [114]. Treatment with mTORC1 inhibitors, such as rapamycin and Torin1, has been shown to rescue the synaptic alterations observed in neurons [114], thus highlighting the utility of hiPSCs as a model for pathology and as a platform for therapeutic screening. 

Organoid models offer a more accurate representation of the complex ontogenesis of the cerebellum, which undergoes a lengthy developmental period. In a pioneering study published in 2015, Muguruma and colleagues developed a protocol for generating cerebellar neurons through 3D cultures of hiPSCs [115]. The cells aggregated and formed a neuroepithelial structure in the presence of specific growth factors and inhibitors. Upon aggregation with mouse EGLs, the neural precursors differentiated into neurons that exhibited morphological and electrophysiological characteristics similar to those of human PCs. GCs, interneurons, and DN neurons were identified in the 3D aggregates, albeit with a low efficiency [115]. The maturation of the neurons obtained through mouse co-culture exhibited characteristics consistent with those observed during the first trimester of pregnancy. In 2021, Nayler and colleagues [116] developed a long-term culture system that allowed neural networks to form and lobular morphogenesis to occur without the need for mouse cells. This was achieved by adapting the organoid protocol using hiPSCs, which introduced *Matrigel*, a solubilized basement membrane preparation, to induce differentiation within a defined microenvironment.

Over a 90-day culture period, all the major cerebellar developmental neuronal cell types were identified, including RL cells, GCPs, and immature PCs. Notably, these cells exhibited low levels of *ALDOC* or *RORA* marker genes, which are indicative of mid to late embryonic cerebellar development [116]. It is noteworthy that certain genes associated with ASD were found to be enriched in the glutamatergic DCN, suggesting that the organoid model may offer insights into the pathogenesis of the disease [116]. 

Recent advances have improved the cerebellar organoid models, allowing the recapitulation of more advanced developmental stages, in particular the establishment of functional neural circuits between GCs and PCs. Atamian and colleagues [117] successfully generated organoids that, after 60 days of culture, exhibited spatial segregation of stem niches reminiscent of the VZ and the RL, including the human-specific RL_VZ_ and RL_SVZ_. Furthermore, Bergmann glial cells, immature PCs, and GCPs, which are characteristic of fetal cerebellar development, were also observed in these organoids. The addition of SDF1, a chemoattractant factor that drives the migration of GCPs, resulted in the identification of organoid regions that resembled the EGL and PCL. At the six-month time point, lamination of GCs and PCs was evident, with both excitatory and inhibitory neurons exhibiting functional spontaneous activity at 60 days and more coordinated firing at six months [117]. Importantly, for the first time, the *in vitro* differentiated PCs closely resembled late midgestational *in vivo* neurons, suggesting the potential utility of cerebellar organoids to explore ASD neurophysiology. Furthermore, expression analysis revealed a significant enrichment of ASD risk genes in different neuronal cell types within the organoids. These included progenitor and mature molecular layer interneurons, immature and mature inhibitory neurons of the cerebellar nuclei, and especially immature and differentiated PCs. 

The advancement of cerebellar organoid models has yielded invaluable insights into the development and functionality of the human cerebellum, while also elucidating the alterations in these processes that predispose individuals to ASD. Furthermore, the use of organoids developed from patient-derived hiPSCs could potentially be useful in elucidating the specific events that initiate the pathology, as opposed to those that are merely associated with it. This would represent a significant advancement in the development of precision therapy. 

**Figure 5:**
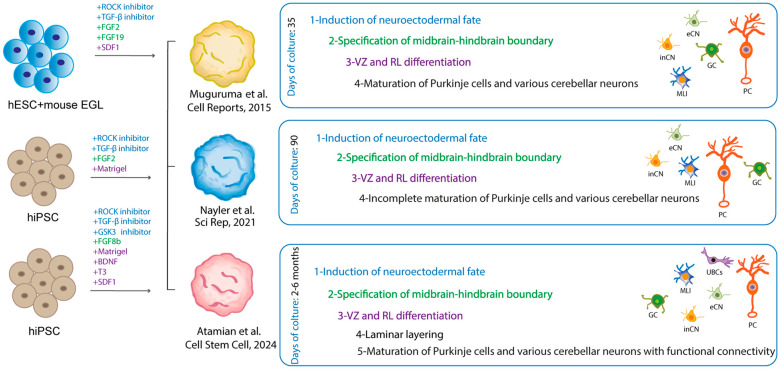
Comparative representation of the cerebellar organoids. The figure presents a comparative analysis of cerebellar organoids derived from different protocols, as illustrated in the indicated studies. The cultured cells and the factors added to the medium to obtain the self-assembling organoid are indicated. The use of fonts with the same colors was employed to indicate the different stages identified in the organoid, along with the factors responsible for them [115,116,117]. The neurons differentiated in the organoids are schematized in the manner indicated in Figure 3.

It is important to note that these models have limitations that need to be overcome in the future. Most notably, there is variability and reproducibility in the protocols used (Figure 5). Moreover, the profiling of non-neuronal cell types, including astrocytes, Bergmann glial cells, microglia, and vascular endothelial cells, in cerebellar organoids must be enhanced. This is because most of ASD-associated genes are expressed in these cells. The resolution of these critical issues will constitute the inaugural stage in the advancement of cerebellar assembloids, the subsequent generation of brain organoids that will permit the combination of multiple brain regions and/or cell lineages in three-dimensional culture [118].

## 7. Conclusions

**Figure 6:**
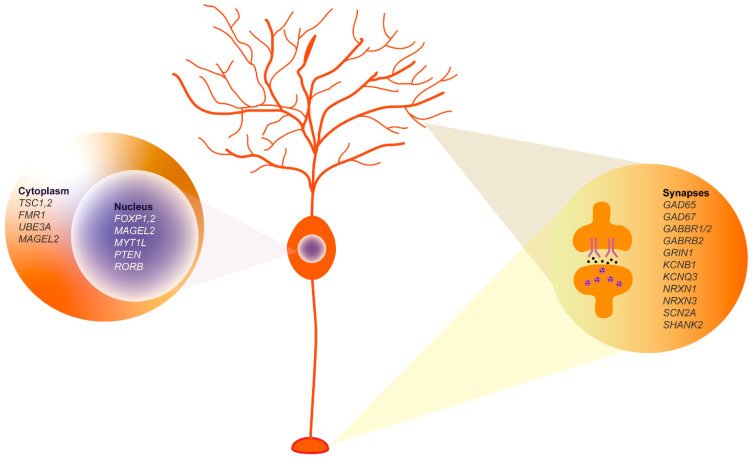
Purkinje cell molecular profile highlighting key ASD-associated genes. The genes shown, derived from extensive database analyses and synthesized conclusions of our review, represent key elements in ASD pathophysiology. **Nucleus**: Genes known to influence transcriptional regulation and neuronal differentiation are shown, including *FOXP1-2*, *MAGEL2*, *MYT1L*, *PTEN*, and *RORB*. **Cytoplasm**: The TSC1, 2, and FMR1 proteins involved in signaling pathways critical for neuronal communication and synaptic function are shown. **Synaptic region**: Includes synaptic proteins such as GAD65, GAD67, GABBR1/2, and SHANK2, among others, which are essential for synaptic transmission and plasticity.

This review highlights the pivotal role of the cerebellum in the etiology and pathophysiology of ASD, a role that has been historically underappreciated compared to cerebral functions. The involvement of this brain area in ASD has led to the proposal of a clinical trial using non-invasive transcranial magnetic or direct current stimulation as a possible treatment for some of the symptoms of ASD in children (such as repetitive behaviors and hyperactivity) (https://clinicaltrials.gov/ NCT04446442). In recent years, significant progress has been made in identifying ASD-associated genes with hundreds of genetic variants implicated in the disorder. These genes modulate essential processes at the cytoplasmic, nuclear, and synaptic levels, thereby influencing the overall functionality and connectivity of the neurons. Figure 6 illustrates the pivotal role of Purkinje cells in the pathophysiology of the disorder, since ASD-associated genes are expressed in distinct compartments of these neurons, particularly during their maturation. Since ASD is a neurodevelopmental disorder, it is essential to know how the affected areas develop. The knowledge of the mechanisms underlying the determination and maturation of the human cerebellum is still at an early stage compared to what is known about other brain areas, particularly the cerebral cortex. An important aspect is understanding why genetic and environmental insults during a critical period of cerebellar development can have broad effects on cognition and behavior. It is clear that the interplay between GCs and PCs is fundamental for the proper refinement of cerebellar circuitry, and it is intriguing that only PCs appear to be susceptible to ASD development, as evidenced by their morphological, functional, and molecular alterations. It is likely that the expression of ASD-associated genes enriched in PCs during the prenatal period [68,69,105] also characterizes other cell types such as glutamatergic and GABAergic DCN or MLIs, which may be affected in the disorder. In particular, MLIs, which are important for modulating PC activity through their inhibitory input, may contribute to changes in excitatory/inhibitory network connectivity driven by altered GABA signaling of ASD-altered PCs.

The elucidation of the genomic, transcriptomic, and proteomic landscape that characterizes ASD has been greatly accelerated by the ability to acquire and analyze a vast amount of data. At the same time, this large body of data must be subjected to rigorous and unambiguous interpretation in order to ensure convergence between basic and clinical research. For instance, it is evident that there are discrepancies between the various databases that collect data on autism-associated genes, which makes it challenging to identify which are the most promising candidates for translational research. In this regard, an interesting pipeline has been recently proposed by Schaaf and colleagues [119].

Recent studies and reviews have highlighted the potential of artificial intelligence (AI) to revolutionize our understanding of ASD. This technology offers insights into the integration and the analysis of vast amounts of genomic, transcriptomic, and proteomic data, as well as the identification of patterns and prediction of disease phenotypes with unprecedented precision [120,121]. The integration of comprehensive genetic data obtained through AI with functional analysis provided by organoids, particularly those derived from patient-specific hiPSCs, will enable researchers to elucidate the multifaceted nature of ASD more effectively and facilitate the development of more efficacious treatments.

Basically, this review highlights the critical role of the cerebellum in ASD and emphasizes the importance of integrating genetic, neurobiological, and modelling approaches to elucidate the complex mechanisms underlying the disorder. Continued research efforts aimed at overcoming current challenges and translating findings into clinical applications have the potential to advance personalized therapies for individuals with ASD.

## Figures and Tables

**Table 1:** List of 43 genes derived by overlap analysis shown in the Figure 1.

ADNP	Transcription factor that may mediate neuroprotective effects in normal growth and cancer
ANK2	Member of ankyrin family of proteins that link the integral membrane proteins to cytoskeleton
ANKRD11	Ankyrin repeat domain-containing protein. Inhibits ligand-dependent activation of transcription
ARID1B	Component of the SWI/SNF chromatin remodeling complex. May play a role in cell cycle activation
ASH1L	Member of the trithorax group of transcriptional activators
ASXL3	Putative Polycomb group protein involved in the transcriptionally repressive state during development
CHD2	DNA-binding helicase that binds to the promoter of target genes, leading to chromatin remodeling
CHD8	DNA helicase that acts as a chromatin remodeling factor and regulates transcription
CTNNB1	Part of a complex of proteins that constitute adherens junctions
DEAF1	Transcription factor that binds to retinoic acid response element. Inhibitor of cell proliferation
DNMT3A	DNA methyltransferase involved in epigenetic modifications
DYNC1H1	Dynein 1. Acts as a motor for the intracellular retrograde motility of vesicles and organelles along microtubules
DYRK1A	Kinase that promotes cell survival
FOXP1	Transcriptional factor regulator of tissue- and cell type-specific gene transcription during development
GIGYF1	Component of gyf family of adaptor protein involved in tyrosine kinase receptor signaling
GRIN2B	NMDA receptor family member involved in excitatory synaptic transmission in the CNS
IRF2BPL	E3 ubiquitin protein ligase involved in the degradation of target proteins in CNS development
KCNQ3	Member of the family of potassium channel proteins
KDM6B	Lysine-specific demethylase and epigenetic modifier during cellular differentiation and development
MBD5	Member of the methyl-CpG-binding domain (MBD) family. Is involved in cell division, growth, and differentiation
MED13L	Component of the mediator complex involved in the transcription of RNA polymerase II-dependent genes
MYT1L	Member of the zinc finger superfamily of transcription factors, found only in neuronal tissues
PHF21A	Component of the BHC complex that represses transcription of neuron-specific genes in non-neuronal cells
POGZ	Plays a role in mitotic cell cycle progression, kinetochore assembly, and mitotic sister chromatid cohesion
PTEN	Phosphatidylinositol-3,4,5-trisphosphate 3-phosphatase involved in tumor suppression
RAI1	Transcriptional regulator of the circadian clock components involved development and neuronal differentiation
RFX3	Transcriptional activator factor that binds DNA, required for cell differentiation in endocrine pancreas development
RORB	Protein of family of orphan nuclear receptors involved in cytoarchitectural patterning of neocortical neurons
SCN2A	Voltage-gated sodium channel subunit, responsible for action potential initiation and propagation in excitable cells
SETD5	Regulator of chromatin and RNA elongation rate; crucial in NSC proliferation and synaptic transmission
SHANK2	Synaptic protein that functions as a molecular scaffold in the postsynaptic density of excitatory synapses
SHANK3	Synaptic protein that functions as a molecular scaffold in the postsynaptic density of excitatory synapses
SLC6A1	Gamma-aminobutyric acid (GABA) transporter responsible for the reuptake of GABA from the synapse
SMARCC2	Component of SWI/SNF neuronal progenitor-specific chromatin remodeling complexes
STXBP1	Syntaxin-binding protein that participates in the regulation of synaptic vesicle docking and fusion
SYNGAP1	Major constituent of the PSD essential for postsynaptic signaling and synaptic plasticity
TAOK1	Serine/threonine-protein kinase involved in DNA damage response and regulation of cytoskeleton stability
TBR1	Transcriptional repressor involved in cortical development, neuronal migration, and axonal projection
TCF20	Transcriptional activator that binds to the regulatory region of MMP3 and thereby controls stromelysin expression
TCF4	Transcription factor involved in the initiation of neuronal differentiation
TLK2	Serine/threonine-protein kinase involved in DNA replication, transcription, repair, and chromosome segregation
TRIP12	E3 ubiquitin-protein ligase involved in the ubiquitin fusion degradation (UFD) pathway and regulation of DNA repair
WAC	Signaling protein involved in cell-cycle checkpoint activation in response to DNA damage

**Table 2:** *RORB*-target genes expressed by PCs and included in the SFARI database.

FOXP1	Transcriptional factor regulator of tissue- and cell type-specific gene transcription during development
FOXP2	Transcriptional factor expressed in fetal and adult brains and involved in development
GABRB2	Gamma-aminobutyric acid (GABA) A receptor mediating inhibitory synaptic transmission in the central nervous system
GRIN1	Subunit of N-methyl-D-aspartate receptors, members of the glutamate receptor channel superfamily involved in synaptic plasticity
KCNB1	Member of the family of potassium channel proteins
KCNQ3	Member of the family of potassium channel proteins
MAGEL2	Gene involved in the terminal differentiation of neurons
MYT1L	Member of the zinc finger superfamily of transcription factors, found only in neuronal tissues
NRXN1	Cell-surface receptors involved in synapse formation in the central nervous system and required for efficient transmission
NRXN3	Cell-surface receptors involved in synapse formation in the central nervous system and required for efficient transmission
RORB	Protein of family of orphan nuclear receptors involved in cytoarchitectural patterning of neocortical neurons
SCN2A	Voltage-gated sodium channel subunit, responsible for action potential initiation and propagation in excitable cells
SHANK2	Synaptic protein that functions as a molecular scaffold in the postsynaptic density of excitatory synapses

## Glossary

**GWAS genome-wide association study:** Observational study of a genome-wide set of genetic variants in different individuals to see if any variant is associated with a trait. GWA studies typically focus on associations between single-nucleotide polymorphisms (SNPs) and traits like major human diseases (commercial genome-wide SNP arrays). This type of study asks if the allele of a genetic variant is found more often than expected in individuals with the phenotype of interest. A key step in most GWA studies is the imputation of genotypes in SNPs not on the genotype chip used in the study.

**WES:** Whole-exome sequencing. A technique for sequencing the protein-coding regions of the genome (exome) unlike the **WGS**, which provides whole genome sequencing. WES has the advantage of lower costs thus allowing the sequencing of a large number of samples.

**Enhancers:** Short sequences of DNA recognized by transcription factors that, by binding upstream or downstream from the start site of a gene, increases its expression, stimulating its transcription.

**Eyeblink conditioning:** A form of associative learning that involves the cerebellum and its circuits. It consists of the ability to associate two sensations that arrive within a very short time of each other. To study this kind of learning, a bright light is shone at the mice, followed a split second later by a puff of air that always causes the mice to blink. After this happens dozens of times, the mice begin to blink earlier, as soon as the light appears, in anticipation of the puff of air.

**TADA method:** A statistical framework based on a hierarchical Bayesian method to narrow the pool of genetic variants generated by high-throughput genome sequencing methods and to establish their relevance to the disease of interest. To identify risk genes, the method includes de novo and rare variants transmitted in family trios (father, mother, and an affected child) and data obtained by case-control studies.

**Protein–protein interaction (PPI) network:** Refers to the dataset based on the physical associations between two or more proteins in a cell or organism. In the network, a node is a protein and an edge is an interaction between two proteins. The hub proteins in the network are those with higher connectivity and therefore more important biological role. With the correlation approach, the PPI network provides functional information about unannotated proteins.

**Induced human pluripotent stem cells (hiPSCs):** Adult somatic cells reprogrammed into stem cells with a self-renewal capacity that can be induced to differentiate into multiple cell lineages in vitro. These cells maintain the genetic background of the healthy and patient donors.

**Organoid:** Self-organized 3D tissue derived from either pluripotent or tissue-resident stem cells, or progenitor or differentiated cells from healthy or diseased tissues. This in vitro model aims to mimic the key functional, structural, and biological complexity of an organ.

**Assembloid:** Culture model resulting from the integration of two or more organoids or an organoid and cells or tissue fragments. Brain assembloids permit the integration of different regions to model cell migration and neuronal projection and connectivity integration. Different cell lineages cultured with organoids permit, for example, the formation of the vasculature in the 3D models.

**Resting state networks (RSN):** In the functional magnetic resonance imaging (fMRI), sets of brain areas that undergo correlated spontaneous slow fluctuations in the blood oxygenation level dependent (BOLD) signal when no tasks are performed. These areas are functionally connected and correspond to the default mode network (DMN) composed of the medial prefrontal cortex, the posterior cingulate cortex, the inferior parietal lobule, the lateral temporal cortex, and the hippocampal formation.

**Anterior intraparietal lobule:** In the posterior parietal cortex, corresponds to the area anterior to the intraparietal sulcus. It contains neurons that respond primarily to the hand postures used to grasp an object.
